# Non-Destructive Measurement of Egg’s Haugh Unit by Vis-NIR with iPLS-Lasso Selection

**DOI:** 10.3390/foods12010184

**Published:** 2023-01-01

**Authors:** Leiming Yuan, Xueping Fu, Xiaofeng Yang, Xiaojing Chen, Guangzao Huang, Xi Chen, Wen Shi, Limin Li

**Affiliations:** College of Electrical and Electronic Engineering, Wenzhou University, Wenzhou 325035, China

**Keywords:** egg, freshness, visible near-infrared (vis-NIR), least absolute shrinkage and selection operator (Lasso), interval partial least square (iPLS)

## Abstract

Egg freshness is of great importance to daily nutrition and food consumption. In this work, visible near-infrared (vis-NIR) spectroscopy combined with the sparsity of interval partial least square regression (iPLS) were carried out to measure the egg’s freshness by semi-transmittance spectral acquisition. A fiber spectrometer with a spectral range of 550-985 nm was embedded in the developed spectral scanner, which was designed with rich light irradiation mode from another two reflective surfaces. The semi-transmittance spectra were collected from the waist of eggs and monitored every two days. Haugh unit (HU) is a key indicator of egg’s freshness, and ranged 56–91 in 14 days after delivery. The profile of spectra was analyzed the relation to the changes of egg’s freshness. A series of iPLS models were constructed on the basis of spectral intervals at different divisions of the spectral region to predict the egg’s HU, and then the least absolute shrinkage and selection operator (Lasso) was used to sparse the number of iPLS member models acting as a role of model selection and fusion regression. By optimization of the number of spectral intervals in the range of 1 to 40, the 26^th^ fusion model obtained the best performance with the minimum root mean of squared error of prediction (RMSEP) of 5.161, and performed the best among the general PLS model and other intervals-combined PLS models. This study provided a new, rapid, and reliable method for the non-destructive and in-site determination of egg’s freshness.

## 1. Introduction

Eggs are essential daily consumptions for people, and provide protein, omega-3 fatty acid, selenium, and vitamins for human diets [1,2]. Their affordable price and rich nutrition make them wide and large consumptions, and they are considered among the most cost-effective sources of animal protein. Eggs have been widely used in the ingredients of foods such as cakes, biscuits, bread, puffed food, and cold drink products, which play the key role of seasoning, additives, fermentation, and emulsification. One of the critical indicators for evaluating the quality of eggs is freshness, and the freshness decrease occurs with the increase of storage time. In general, consumers regard the decline in egg freshness as a lack of quality [3]. There are many indicators to express the freshness of eggs, such as haugh unit (HU), weight loss rate, yolk coefficient, pH, air cell height and egg shape index, etc. Among these, HU is an accepted commercial and research standard for evaluating the freshness quality of chicken eggs. The reduction in egg freshness can be explained by the marked changes in carbohydrate moieties of ovomucin, in disulfide bonds of ovomucin, or in ovomucin–lysozyme interaction. Furthermore, through the shell pores, the gaseous exchanges with the ambient (H_2_O and CO_2_), and it leads to the air pocket volume increasing. Therefore, the changes occur not only in the internal physical and chemical indicators, but also on the shell surface of egg. However, the traditional physical-chemical methods to measure these changes have some disadvantages, such as time-consuming, destructive, and affecting secondary sales. Therefore, it is urgent to develop a rapid non-destructive detection method to identify and classify the quality of eggs.

In modern egg production and process, how to quickly and effectively detect the freshness of eggs has become more important. Several optoelectronic sensing technologies were employed to measure the egg’s freshness, such as Fourier transform infrared spectroscopy (FT-IR) [4], hyper-spectral imaging approach [5], Raman spectroscopy [6], acoustic spectroscopy [7], dynamic weighing and image descriptors [8]. A significant amount of research reports showed the potential of spectroscopy to predict the quality of eggs [2,4,5,6,8,9,10,11]. As a vibrational spectrum, near-infrared spectroscopy can express information on the egg’s external and internal attributes [4,6,10,11,12,13,14,15]. In recent years, small, low-cost, handheld, and ergonomic visible (Vis) or near-infrared (NIR) fiber spectrometer devices have been commercially available. In contrast with the benchtop Fourier-transform infrared (FT-IR) or NIR devices, fiber spectrometer can be implemented on portable devices or production lines for in situ measurement at different sites in the supply chain of the egg industry, and has been proven its success in detecting the egg’s freshness [10,13,14,15,16].

Although these reports have achieved the satisfying results with multivariate modeling methods, they are mainly on the basis of the NIR spectrum with the long wavelength, and the short wavelength NIR (SW-NIR) spectrum less than 1000 nm usually obtains a little worse predictive performance for egg’s freshness [4,14,15]. As known that the SiO_2_ detector for SW-NIR signal is not expensive and the practical applications prefer low-cost designs [17]. Thus, the spectrum in the range of visible to SWNIR should pay more attention to the measurement of egg’s freshness. Partial least square (PLS) was used most to develop the regression model to fit the attributes of samples, and interval PLS (iPLS) was proposed on the basis of piecewise modeling of spectral intervals and determination of the most informative interval range [18,19]. However, in a spectrum that is divided into several intervals, it is not possible to have only one interval correlated with the attributes while the rest intervals are not. If other intervals are integrated to develop the model, can the accuracy of model be improved? Furthermore, in some solutions, only using a single modeling method may make the model’s prediction unsatisfied with low accuracy or poor robustness in the prediction stage, even with complex non-linear modeling approaches [12,20,21]. In order to solve the problem of inaccurate prediction of a single model, some literatures adopt a fusion strategy to integrate several member models into a fusion model, which can further improve the performance of model and achieve a good result in practical applications [20,21,22,23].

In this work, a fusing strategy was proposed based on the interval partial least square member model, and the least absolute shrinkage and selection operator (Lasso) [24,25] was employed to act as the interval selection and integrate a fusion model from the selected iPLS models. By the above analysis, this work is to explore the application potential of vis-NIR spectroscopy in the measurement of egg freshness. Specifically, it is divided into several sub-topics: (1) collecting and analyzing the vis-NIR spectra of eggs; (2) optimizing the spectral pretreatments and developing PLS and iPLS models; (3) establishing iPLS-Lasso fusion models based on iPLS member models, other interval-based models [15]; (4) systemically comparing the performance of models and identifying the best one.

## 2. Materials and Methods 

### 2.1. Sample Preparation

Brown eggs (bens were about 200 days old) were provided by a local poultry farm (Wenzhou City, China) within 24 h after delivery. In this case, 105 eggs with intact and clean eggshells were pre-selected for the subsequent experiments. Another 20 eggs were used to replace artificially damaged eggs or eggs with chemical outliers. Eggs were stored from 1 to 13 days, respectively, with 20 ± 2 °C. In the period of the storage, a batch of 15 eggs were randomly picked out every two days for spectral acquisition, and then were cracked for destructive detection. 

The weight of an egg was weighed by an electronic scale with a precision of 0.01 g, and the short and long axis were measured by a vernier caliper with a precision of 0.02 mm. As a result, a total of 105 eggs were measured overall.

### 2.2. Vis-NIR Spectroscopy Measurement

**Figure 1:**
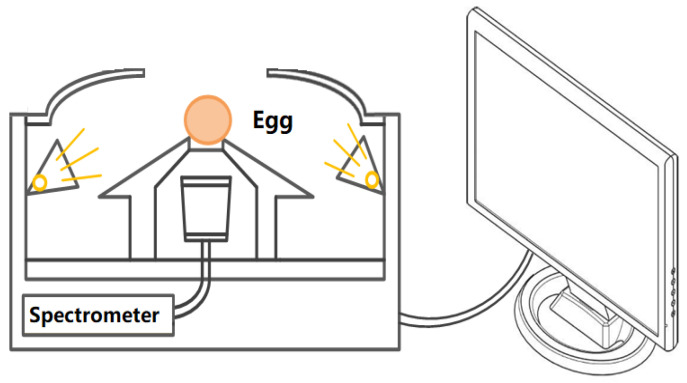
The schematic diagram of the near infrared spectrum acquisition system of eggs.

Spectra of eggs were collected by a developed portable prototype (Figure 1), which had been described in previous work [26]. It was consisted of a Maya2000pro fiber spectrometer (Ocean Optics Inc., Dunedin, FL, USA) with a range of 550~982 nm, illuminating and acquiring accessories. Six tungsten lamps (12V 20W, MR11, Osram, Munich, Germany) were arranged equidistantly with a small upward angle around the supported holder to radiate on the egg. In practical applications, it is cumbersome to continuously measure the diffuse-reflective spectroscopic signal at multiple sites, and thus the transmittance mode is preferred for spherical objects. In the transmittance mode, the radiation must be sufficient enough to penetrate the object. In this work, three parts of radiation onto sample were ingeniously designed. The main radiating part directly delivered onto the surface of sample is the lighted lamps, and the other two parts were the divergent lights that were reflected onto samples by two reflective accuracies (inner surface of lamp chimney and upper surface of the supported holder). For the medium size of fruit or agri-product, such as pear, apple, or orange, six lamps are turned on, and for the small size and high spectral penetration, such as egg, three interval lamps are turned on. The circular gasket attached to the top of the supported holder can prevent stray light into the probe of the spectrometer. 

Three main parameters of spectral acquirement were set the integration time of 100 ms, the smooth boxcar window of 6, and the acquisition time of 4 [26]. Before spectral acquisition, lamps were turned on for preheat at least 10 min. The egg to be tested was placed on the supported holder, and the spectral sampling points were close to the maximum diameter of egg’s transverse. The dark spectrum and reference spectrum were pre-stored, and the transmission signal (intensity) was automatically converted to transmission (T%) through the software control. For each egg, three spectra were acquired at different sampling points, where the egg was rotated every 120° around the long-axis of the egg. From a total of 3 spectral readings, the averaged spectrum was calculated for each egg. 

### 2.3. Measurement of Egg’s Freshness

After spectral collection, weight loss rate (WLR), haugh unit (HU), and yolk coefficient (YC) was measured to describe the freshness of the brown egg. An egg was weighed and broken onto a horizontal flat surface to measure the thick albumen height (H), yolk’s diameter, and height. YC was expressed as dividing the center height of the yolk by its diameter. WLR was defined as the ratio of weight loss to the arrival weight. HU was calculated by Equation (1) referred to previous work [27].

(1) $$HU=100\cdot \log(H-1.7\cdot W^{0.37}+7.57)$$

where *W* is the weight of egg. *H* is the average value of four measurements, carried out on different sites to acquire the thick albumen height at a distance of 10 mm from the yolk ([2,28]).

### 2.4. Multivariate Data Analysis

#### 2.4.1. Spectral Pretreatments

Three main pretreatments of the first derivation with Savitzky-Golay moving smooth (D1st S-G), multiplicative scatter correction (MSC), and standard normal variate (SNV), are used to remove the unwanted noises and improve the spectral quality. 

#### 2.4.2. Interval Partial Least Square (iPLS)

Interval partial least square (iPLS) is also a PLS modeling method just on the basis of one spectral interval, which was commonly regarded as the informative spectral region related to the analyte [18]. Thus, in some cases, iPLS is used to select the featured spectral interval and eliminate the influence of multi-collinearity between the spectral intervals. The basic principle for iPLS modeling method is: firstly, the full-band spectrum of *p* wavelengths is divided into *n* disjoint intervals with equal width (*p*/*n* wavelengths); next, a series of local PLS regression models are established on each spectral interval, respectively; lastly, the predicted residual sum of squares (PRESS) from each local PLS model are compared, and the best iPLS model is picked out with the lowest RMSECV. With a suitable width and position of spectral interval in the spectrum, iPLS can obtain a comparable predictive performance than the full PLS model.

#### 2.4.3. Selections by Lasso

Least absolute shrinkage and selection operator (Lasso) is a matrix-sparse method for compressing the spectral information from the high-dimensional dataset. It was proposed by Tibshirani [24,25] to reduce the dimensionality of inputs based on a regularization technique, whose function is to minimize the residual sum of squares subject to the sum of the absolute value of coefficients that were less than a constant. Lasso uses the regularization function (as Equation (2) shown) to penalize the input variables with less information, making the associated inputs tend to zero. Hence, if the regularization was applied to member models, it could select those informative models which were not penalized and performed as the model selection. Lasso regularized the estimated parameters (weighting and bias) and minimized the following cost function:

(2) $${\hat{\beta }}_{lasso}=\underset{\beta }{\arg\min}\{\sum_{i=1}^{N}{\left(y_{i}-\beta_{0}-\sum_{j=1}^{p}{\hat{y}}_{ij}\beta_{}\right)}^{2}+\lambda \sum_{j=1}^{p}\left|\beta_{j}\right|\}$$

where, ***N*** is the number of observations, and ${\hat{y}}_{ij}$ is the output of member model. ***y_i_*** is the response at observation ***i***. ***λ*** is a nonnegative regularization parameter corresponding to one value of Lambda. The parameters ***β*_0_** and ***β*** are scalar and ***p***-vector, respectively. With re-weighting attributes for the potential indicators, Lasso can be derived from its utilization to fuse member models into a fusion model. It can weigh the importance of member models and select these with large weighting values for the final fusion model. As a result, Lasso can eliminate some irrelevant variables which have little influence on the final model and simplify the complexity of the model’s structure.

In this study, the main aim of using the Lasso method is to reduce the number of spectral wavenumbers and select the valuable information of spectral intervals. At the same time, Lasso is also a linear regression to fit the dependent variables into a fusion model (iPLS-L), and the potential variables are designated the output of iPLS member models. The main parameters, ‘CV’ of 5-fold cross-validation, ‘Lambda’, were used to estimate the mean squared error and optimize the valuable variables in the Lasso fittings. The best combination of the selected variables is automatically determined by the minimum ‘LambdaMinMSE’ in the structure of Lasso output. If the coefficients of some variables (i.e., the iPLS member model) are 0, the corresponding is discarded, and the purpose of initially screening the best member model combination is achieved. 

**Figure 2:**
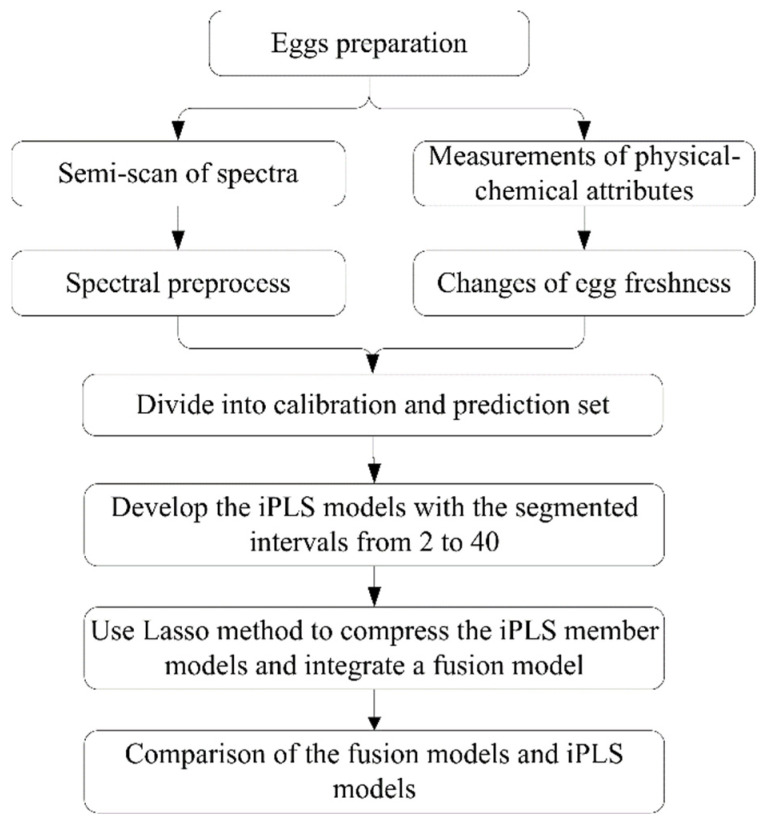
The workflow of the iPLS fusion model by Lasso method.

The flow chart of the Lasso fusion model is shown in Figure 2. The iPLS models were developed with the changes in the number of spectral intervals from 2 to 40. If the number of intervals is 1, it means that the iPLS model is equal to the full-spectral-based PLS model, and this exists only one member model, which is also the iPLS-L fusion model.

#### 2.4.4. Estimation of Model’s Performance

RMSE (root mean squared error), *r* (correlation coefficient), and bias are the commonly used parameters in the regression models. A well-calibrated model usually has a small RMSE and bias, and a large *R* (left-tending toward 1). The derived parameters from RMSE, RMSECV in the cross-validation stage, and RMSEP in the prediction stage were proposed to intuitively observe the predictive error of the developed models. Generally, a qualified calibration model should have small RMSECV and RMSEP, but a slight difference exists between them to avoid under-fit or over-fit. In fusion models, RMSECV and *r_cv_* (correlation coefficient of cross-validation) are calculated from the actual values ***y*** and the value $\hat{y}$ predicted by the fusion model ***F(x)*** at the cross-validation stage of each member model. Similarly, RMSEP and Rp (correlation coefficient of prediction) are calculated in this way at the prediction stage.

### 2.5. Software

In this work, matrix calculations were conducted in MATLAB software (R2018a, Math Works Inc., Natick, MA, USA). The iToolbox was employed to operate the PLS algorithm [18]. The fusion codes were programmed referring to the above formulas.

## 3. Results and Discussion

### 3.1. Changes in Egg’s Freshness

**Figure 3:**
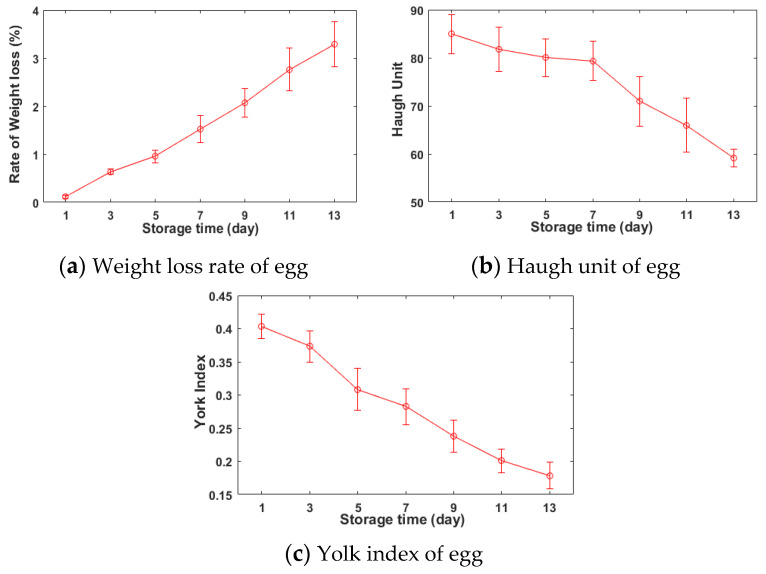
The changes of the freshness of egg during the storage time.

The wider the span of the chemical attributes of egg samples, the better it is for the development of the calibration model, and thus the storage time was suggested a relatively long period to differentiate the freshness of eggs. During the storage, the inner moisture and carbon dioxide (CO_2_) penetrate through the eggshell, causing the loss of egg weight, the increase of acidity, and the change of albumen texture [2]. The thickness of the albumen layer is impacted by changes in ovomucin-lysozyme interaction during the aging process, and this change can be used to assess the freshness of egg by HU [27]. Thus, the HU is about the internal changes of compositions and protein structures. The physical -chemical attributes of the grouped eggs, which were assigned with a series of different storage times, were calculated the averaged/standard deviation values. The trend graphs of HU, yolk index, and weight loss rate were plotted with the change of the assigned days in Figure 3.

It can be observed that with the increase of the number of storage days, the value of the weight loss rate (Figure 3a) increased synchronously, while values of HU (Figure 3a) and yolk index (Figure 3b) decreased gradually. For the freshness of eggs, the higher the HU and yolk index, the better. The lower the weight loss rate, the better. Since the differences between individual eggs, the freshness of individuals changed inconsistently during the storage. Meanwhile, at the beginning of this experiment, it cannot be guaranteed the quality of eggs to be consistent, and thus the standard deviations of egg’s indicators are changed with corresponding to the storage times. The changing tendency of egg’s freshness in this work is roughly consistent with previous literatures [6,10]. With the increase of storage time, the value of HU decreases gently from 85 on the first arrival day to 79 on the 7th storage day from the first four measurements (regarding the grade of AA), and then drops suddenly to 70 on the 9th storage day (the grade of A) from the fifth measurement. At the same time, the decrease of the standard deviation in the grouped eggs indicates the egg individual consistently turns stale, and the overall freshness begins to decline. As the increase of the storage time, the differences in the weight loss rate between the grouped eggs turn to be larger, which also indicates the freshness of eggs turns to be inconsistent, and the loss of water influences the freshness of egg.

Referring to the previous studies, some factors such as temperature, storage time, humidity, and airflow velocity, mainly impact the quality of eggs [2,11]. In this work, the experimental eggs were stored in the same constant conditional incubator, and the controlling factor was just the storage time.

### 3.2. Physical Parameters of Eggs

**Figure 4:**
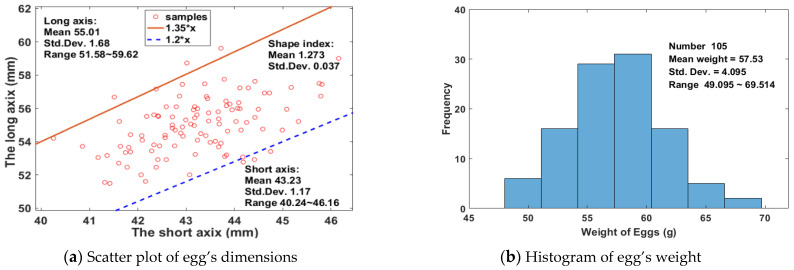
The distribution of basic information of the experimental eggs.

In the national standard poultry egg grade, the egg shape index is stipulated in the range of 1.3 to 1.35, which is expressed as the ratio of the long axis to the short axis. Although the egg shape index does not affect the eating value of egg, it was significantly correlated with the variety, the hatchability, and the breakage rate of egg. In this work, samples were dimensioned with a short axis of 40.24~46.16 mm and a long axis of 51.48~59.62 mm, respectively, the mean of egg shape index was 1.273 with a standard deviation (Std. Dev.) of 0.037, and over 94% of samples were distributed in the range of 1.20 to 1.35, as shown in Figure 4a, in which are the slopes of two lines passing through the origin (Figure 4a). The weight of eggs were normally distributed in the range of 49.095~69.514 g on the arrival day, with an average of 57.53 g and a standard deviation of 4.095, as shown in Figure 4b.

Before calibrating the model, the abnormal samples (i.e., outliers) should be eliminated first to avoid the inappropriate calibration of the regression model. Typically, the difference of 5% is a significant level of detection. After detection, there are two spectral abnormal samples and no concentration abnormal samples. After removing those two abnormal samples, the eggs were randomly divided into two subsets with a ratio of 2:1. As a result, 69 samples were put into a dataset called Calibration set which is used to develop a regression model, and the rest 34 samples were in the Prediction set which is used to test the robustness of the above developed model. In this work, the reference values were the HU of eggs. Observed in Table 1, the mean, the SD, and the CV in the Calibration set were close to each in the Prediction set. Their range of referred values were basically the same. Through above the distribution of Physical (dimensions, weight) and chemical (yolk index, HU, weight loss rate) attributes of eggs, it showed that the selected samples were representative and widely distributed.

### 3.3. Spectra of Eggs

**Figure 5:**
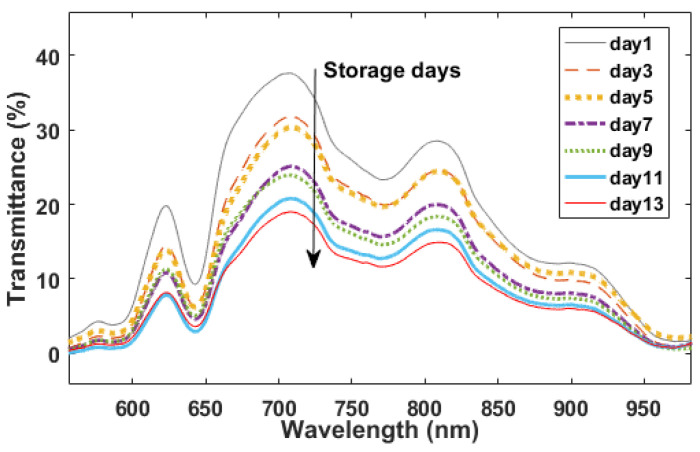
The original visible-near-infrared spectrum of eggs.

The averaged spectra of each grouped eggs in the spectral range of 550–985 nm for different storage days are presented in Figure 5. The trend of these spectra were basically consistent, and the intensity of all spectral region was different with a decrease tendency responding to the storage days. Compared to the diffuse reflectance mode, the transmittance mode of spectral measurement can more effectively characterize the vibrational spectral information of the internal quality of eggs, and the visible-short near-infrared spectroscopy used in this work (550–985 nm) has a stronger penetration ability of agri-products than the other region of near infrared spectroscopy (900–2526 nm). The decline in the freshness of eggs is actually some change in the internal chemical components of egg, such as the saturated fatty acids in the yolk, moisture content, protein structure, and loss of CO_2_. Thus, the penetrated spectrum involves these changes in chemical components, and it can be acted as an indicator of the degree of freshness change. Different from infrared spectroscopy, the visible-short near-infrared spectroscopy used in this work has a small number of fingerprints that can reflect the specific functional groups.

There are some valleys around 645 nm, 770 nm, 880 nm, and 970 nm involved in the profile of egg’s spectrum, indicating the absorption of radiant energy by special functional groups of components. The valleys around 770 nm and 970 nm are referred to associate with the third overtone of O-H group, and the difference between the intensity of spectral regions is caused by the loss of water through the eggshell during the aging process [5,11]. The overlapped valley at the spectral region of 770–790 nm likely has the relation with 2xN-H stretching +2x amide I (protein) or N-H stretching third overtone (ArNH_2_), and displacement in absorbance value can be associated with changes of structural protein during the storage time [2,10]. Other valleys in egg’s spectra are the results of the overlapped or the combination absorptions of the H-contained groups (C-H, O-H, S-H, N-H stretching), such as moisture, proteins, fatty acid, and CO_2_ et al. Obviously, by naked-eye the spectral absorptions are correlated to the functional groups in sample’s components, but the component’s concentration could not be given out through direct observation of NIR spectral profile due to its severely overlapped information and multivariate data modeling analysis is needed.

### 3.4. Spectral Preprocess for PLS Models

Three common preprocess methods, including standard normal variable transformation (SNV), the first derivative with Savitzky-Golay filter of 5 points and 2 degrees (D1st+S-G), and multivariate scattering correction (MSC), were used on the semi-transmitted spectra in the PLS full-spectral modeling process of this work. Due to the uncertainty of both ends of the spectral signal caused by the manufacture of the photosensitive chip, the ends of spectral regions were cropped out and eventually 2041 spectral variables remained for subsequent modeling analysis.

The general PLS models were constructed on the basis of the whole region of the different preprocessed spectra, respectively, with cross-validation in optimizing the number of latent variables (LV), which was initially set to the max of 20. The optimal number of LV is determined according to the minimum of RMSECV. Table 2 shows the statistical result of the PLS model with different spectral preprocesses for HU.

By comparison of *r*, Bias, and RMSE, in these developed models, the full-spectral-based PLS model with the pretreatments of SNV and MSC, got slightly better performances than that without any pre-processed method at the calibrating stage. Oppositely, the performance of PLS model with S-G D1st pretreatment turned worse. It may be explained that the differential operation not only removes the uninformative background signals, but also magnifies local noise involved in the spectra, resulting in larger deviations [29]. Although the approximate performances of PLS model with SNV and MSC correspond to the parameters of r and RMSE, the predictive performance performed close to each, and just the smaller bias by SNV and MSC. Thus, it can be concluded that these proposed pretreatments have limited information on the egg’s spectra in this work, and no pretreated spectra were used in subsequent analysis.

### 3.5. Development of iPLS Models

By above optimization of the spectral preprocesses, the original spectra were used to regularize the spectra before calibrating the iPLS model. In the development of iPLS models, the full spectral region was segmented equally into a series of intervals from 2 to 40 in sequence, and the corresponding PLS model was constructed based on each sub-interval, respectively. For each spectral segmentation, the optimal spectral sub-interval is determined at the cross-validation stage according to the minimum principle of RMSECV among these developed iPLS models, and then the predictive parameters (especially the RMSEP) of iPLS were estimated in the prediction set.

**Figure 6:**
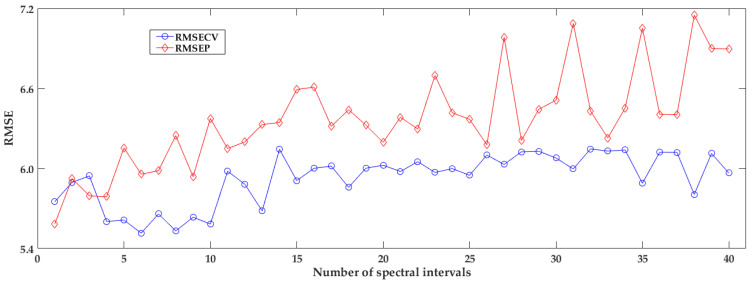
The result of iPLS models with different number of spectral intervals.

The statistical result of iPLS models with different spectral intervals is plotted as Figure 6 shown. It can be seen that the RMSECV and RMSEP are varied and turn to be large with increasing the number of spectral intervals, and the values of RMSEP are larger than that of RMSECV in most intervals. Obviously, different interval has an impact on the performance of iPLS model. Different interval numbers must lead to different divisions of the full spectral matrix. If a sub-interval contains more useful spectral information and less noise, the performance of the corresponding iPLS is better. The bigger the number of spectral sub-intervals, the less the number of spectral variables in each sub-interval. If the number of intervals changes, both spectral bands and the number of spectral bands in sub-interval will be adjusted. This must lead to sub-intervals with different signal-noise ratios. Therefore, the performances of these developed iPLS are fluctuated with the interval number, especially the number of sub-intervals reaches more than 27, and the fluctuation turns to be large. While the number of intervals ranged 4 to 10, iPLS obtained a relatively smaller RMSECV compared to other divisions. In this solution, when the number of spectral sub-intervals is 6, the RMSECV is the smallest among all iPLS models, and it is considered the best with a RMSEP of 5.757, but it still does not perform better than the full-spectral-based PLS model. 

### 3.6. Lasso Selection and Regression

With a specific division of the spectral wavelengths, all iPLS models are the candidates, and their outputs are taken as the member models for the Lasso process. In above analysis of iPLS models, their performances varied with the different sub-intervals and the number of spectral sub-intervals. Thus, under each sub-interval’s division, Lasso linear regression model was constructed between the corresponding attribute of HU and the cross-validated outputs of the above series of iPLS member models, whose intervals were the divisions of 2 to 40 for the spectral region. In the Lasso regression, the tuning parameter ***λ*** controlled the degree of coefficient’s restraint and let ***λ*** be large enough to make some coefficients zero. In order to avoid the interference of artificial preferred settings, the average and standard error of sum squared residual (SSR) in the Lasso regression models were cross-validated with 5-fold in the calibration stage. In this work, the tuning parameter ***λ*** was adaptively obtained by obeying the rule of a minimum of RMSE in the developed Lasso model.

**Figure 7:**
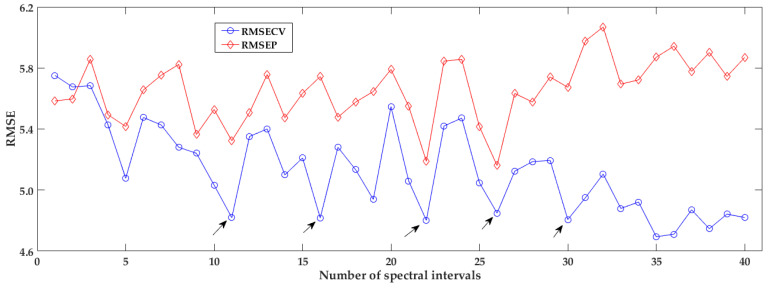
The result of iPLS-Lasso models with different number of spectral intervals.

After Lasso fitting, some coefficients of inputs are exactly zeros or trend to zeros, meaning less contribution to the Lasso regression model, and as result it acted as the variable selection function. The developed Lasso regression model was labeled as iPLS-Lasso model, and the *i*-th model was regarded as iPLS-L*i*. The statistical parameters for estimating the performances of these fusion models were counted, especially the RMSECV and the RMSEP, and were plotted in Figure 7. It can be seen that the values of RMSECV and RMSEP changed with the number of spectral intervals, similar to the performances of iPLS model as changing the number of intervals. The overall trend RMSECV in iPLS-Lasso model is decreasing, while the RMSEP trends to increase, until at the division of 35 intervals their fluctuations tend to be stabilized. Among these fusion models, they all performed better than the general PLS model, which was the first fusion model (considered as the iPLS-L1). Some fusion models obtained a fairly low RMSECV of less than 5.0 in the range of intervals number 5~30, and they were the 11th, 16th, 22th, 26th, and 30th fusion models, respectively. When the number of intervals is divided into more than 30, the iPLS-Lasso fusion model got a smaller RMSECV at the calibration stage, but the RMSEP in the prediction stage turned to be larger, indicating that more intervals involved in the Lasso regression model might result in over-fitting. The predictive performance should also be considered in selecting the fusion model, not just the cross-validating performance.

### 3.7. Comparison and Discussions

Table 3 shows the statistical results of different types of regression models for HU of eggs, including the general PLS model based on the full region spectra, the interval PLS model based on the 4th interval at the division of 6 intervals, five fusion models(iPLS-L) at different divisions of intervals, the stacked fusion model (PLS-s1) integrated from all iPLS member models at the division of 26 intervals, and the PLS model (PLS-s2) based on the selected intervals in the iPLS-L26 fusion model. In the stage of establishing the PLS^a^ model, because it did not select any useful spectral wavelengths from the whole spectral region, which might involve some noises that caused the model to be unreliable, and weakened the predictive performance with an RMSEP of 5.584 in the prediction set. In the development of iPLS model, the spectral region was attempted to divide into 2 to 40 intervals, and on the basis of each interval a series of PLS models were systemically built to compare their performances. When the spectral region was divided into six intervals, PLS built on the 4th interval obtained the best performance than others, and the RMSEP in the prediction set was 5.757. By comparison with PLS, iPLS just used 16.67% spectral wavelengths (about 340 variables) in the range of 777–848 nm, where it was the informative spectra to reflect the internal attributes of samples in previous work [10,11], but the limited spectral region limited their access to more spectral information of inner compositions [18], and thus it performed not better than the PLS model. 

In the developed fusion models, all iPLS models at each division of intervals were constructed to obtain their outputs, and then Lasso was used to act as model selection and regress these outputs into a linear model. At the cross-validation stage, several fusion models that were the 11th, 16th, 22th, 26th, and 30th iPLS-Lasso, respectively, obtained the acceptable RMSECV, and they were listed to compare their predictive performances. Although their RMSECV were low and closed to each, their RMSEP were different in the prediction set. In these five fusion models, the 22th and 26th fusion models performed better than the other three, and acquired lower RMSEP of less than 5.2. Although the 16th and 30th fusion models got a better performance with a lower RMSECV at 4.824, at the prediction stage they performed worse than others with an RMSEP of larger than 5.641. The RMSE of these two fusion models at calibration and prediction stages were quite different, and thus it was inferred that they were probably over-fitted and were not suitable to be the final calibration model [30]. Taking a close look into the 22th and 26th fusion models, the iPLS-L22 model employed more than half spectral wavelengths to be constructed while the iPLS-L26 model used 38.46% of spectral wavelengths to obtain a close performance. 

**Figure 8:**
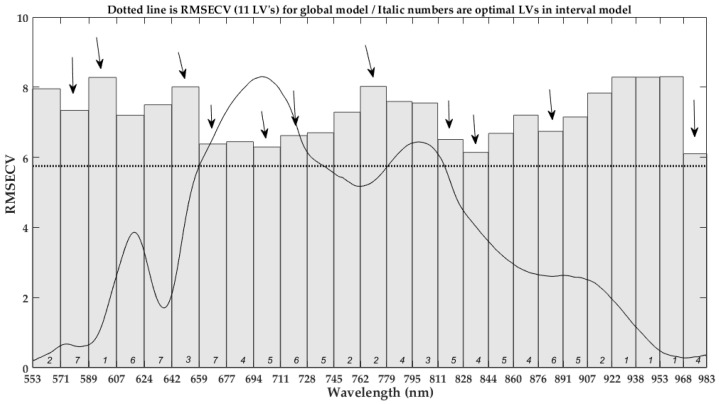
The selected intervals in iPLS-L26 fusion model. The downward arrows indicates the selected intervals by the Lasso regression in the present division of spectral intervals.

At the spectral division of 26 intervals, 10 intervals were selected for the fusion model to develop the iPLS member models by Lasso selection, as Figure 8 shown. The height of bar indicates the RMSECV in the corresponding interval-based iPLS model, and the dotted line shorts for the full-spectra-based PLS model. All iPLS models performed not better than the PLS model, and the 17^th^ iPLS obtained the lowest RMSECV of 6.284 among these intervals in the process of optimizing iPLS model, as Figure 7 depicted. The selected intervals distributed over the entire spectral region, and their distribution was relatively dispersed. They were distributed in the spectral range of 642–728 nm, 762–779 nm, 811–844 nm, and 968–983 nm, respectively, and these spectral regions related to 2xN-H stretching +2x amide I (protein) or C-H, N-H stretching third overtone (ArNH_2_) [5,19,29], as described in Section 3.3. However, the same combination of the above selected spectral intervals was used to construct the PLS-s1 model, which is equal to the synergy interval PLS but without optimization of the interval’s combination [18]. Its performance turned worse with an RMSEP of 5.595, close to the optimal PLS model based on the full spectral region. It indicates the selected intervals contain useful spectral information about the inner compositions, but they are not suitable to directly develop the calibration model [18,31]. Furthermore, the stacked iPLS model (PLS-s2) was developed as descripted in the study [12,19], and it acquired the worst performance among all models.

**Figure 9:**
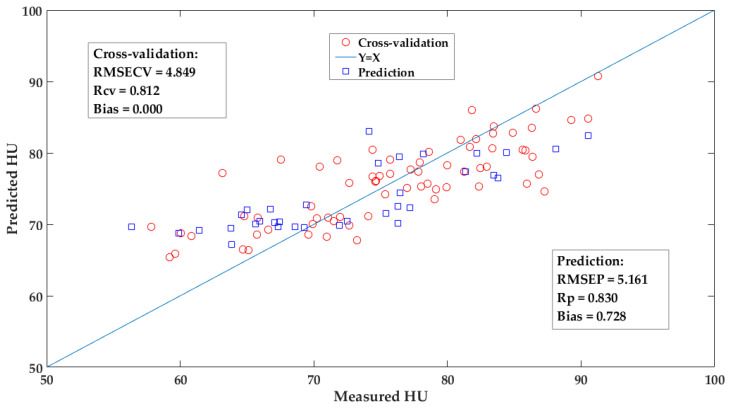
The scatter plot of the measured versus the predicted values by iPLS-L26 fusion model for egg’s HU.

Among these listed models, from the evaluation of the predictive performances, the fusion models performed the best, followed by PLS model, iPLS model, PLS-s1 model, and the worst is the PLS-s2 model. Obviously, due to the limited informative spectra, the optimized iPLS model could not obtain a better performance to some degree than that of the PLS model. While, by the Lasso regularization the fusion model was integrated by several iPLS member models, which usually were the informative spectral intervals, and obtained a relatively low RMSECV with a promotion of 7.6% compared to the PLS model. The coefficients of these selected iPLS member models followed the regularization of Lasso, whose rule was different from other fusion models and they could be attempted in the further study [12,20,22,23]. Figure 9 shows the scatter plot of measured versus the predicted values by the iPLS-L26 model for the egg’s HU. Some samples were not well predicted, especially the eggs with low values of HU, and these were not fresh. Compared to other studies, near-infrared spectroscopy with long wavelength rather (i.e., 1000–2526 nm), Raman or infrared spectroscopy usually acquire better or close accuracy for predicting the HU of egg according to their finger-prints which can reflect the structures of specific functional groups [4,6,10,11]. 

## 4. Conclusions

This study aims to explore the potential application of vis-NIR spectroscopy in non-destructive determination of egg’s freshness, which was modeled by the iPLS member model combined with Lasso selection. The Lasso method is used to compress potential member models, which can reduce the number of spectral wavelengths and simplify the complexity of modeling. Compared to the general PLS model, the proposed iPLS-Lasso fusion models have a better performance. By optimizing of the number of spectral intervals, the iPLS-L26 fusion model obtained the lowest RMSEP of 5.161 with a promotion of 7.6% for egg’s HU. Although the vis-NIR with short wavelengths is not enough to represent the spectral information for the internal quality of eggs, the subsequent modeling strategy shows the acceptable application potential in the detection of egg’s freshness, and it has the advantages of fast, timely, and low cost.

## Figures and Tables

**Table 1:** The statistical results of Eggs’ yolk index and haugh unit in different sets.

Measurements	Datasets	SN ^a^	Range	Mean	SD ^b^	CV ^c^ (%)
Haugh unit (HU)	Calibration set	69	57.8~91.3	75.2	8.6	11.44
	Prediction set	34	56.3~81.6	73.9	8.1	11.02

SN ^a^: the number of samples; SD ^b^: standard deviation; CV ^c^: coefficient of variation.

**Table 2:** The performance of PLS models with different pre-processes for egg’s HU.

Detection Indicator	Pre-Process	LVs	Calibration Set			Prediction Set		
			RMSECV	*r_cv_*	Bias	RMSEP	*r_p_*	Bias
Haugh unit	MSC	8	5.673	0.737	0.000	5.589	0.757	0.295
	SNV	8	5.672	0.737	0.001	5.587	0.757	0.276
	D1st+S-G	7	5.915	0.699	−0.106	6.107	0.714	1.277
	None	11	5.751	0.739	−0.042	5.584	0.757	1.427

Note: MSC: Multiplicative Scatter Correction; SNV: Standard Normal Variate; D1st+S-G: First deviation with S-G smoothing; LV: the number of latent variables in partial least squares model.

**Table 3:** The performance of iPLS models with different intervals for HU of eggs.

Models	The Selected Intervals	Calibration Set			Prediction Set		
		RMSECV	*r_cv_*	Bias	RMSEP	*r_p_*	Bias
PLS ^a^	1/1	5.751	0.739	−0.042	5.584	0.757	1.427
iPLS ^b^	4^th^/6	5.517	0.744	0.001	5.757	0.73	−0.002
iPLS-L11 ^c^	10/11	4.829	0.812	−0.000	5.323	0.808	1.881
iPLS-L16	5/16	4.824	0.814	0.000	5.744	0.728	−0.159
iPLS-L22	14/22	4.805	0.839	0.000	5.189	0.827	0.745
iPLS-L26	10/26	4.849	0.807	0.000	5.161	0.832	0.728
iPLS-L30	8/29	4.815	0.832	0.000	5.641	0.751	0.594
PLS-s1 ^d^	10/26	5.821	0.736	−0.185	5.595	0.755	1.309
PLS-s2 ^e^	26/26	6.246	0.712	0.120	6.707	0.734	1.418

Note: PLS ^a^: the general partial least squares regression model with the optimal LV of 11; iPLS ^b^: the interval partial least squares regression model with the 4th intervals at the division of 6; iPLS-L ^c^: the fusion model on the basis of iPLS member models by Lasso regression; PLS-s1 ^d^: the developed PLS model with optimal LV of 6 based on the spectra selected from the iPLS-L26 fusion model; PLS-s2 ^e^: the stacked fusion model based on each iPLS member model at the division of 26 intervals.

## Data Availability

Data is contained within the article.
